# Novel immune-modulator identified by a rapid, functional screen of the parapoxvirus ovis (*Orf virus*) genome

**DOI:** 10.1186/1477-5956-10-4

**Published:** 2012-01-13

**Authors:** Michael J McGuire, Stephen A Johnston, Kathryn F Sykes

**Affiliations:** 1 Center for Innovations in Medicine, The Biodesign Institute School of Life Sciences, Arizona State University, 1001 McAllister Ave, Tempe, AZ 85287-5901, USA; 2 Division of Translational Research Department of Internal Medicine University of Texas Southwestern Medical Center 5323 Harry Hines Boulevard Dallas, TX 75390-9185, USA

**Keywords:** adjuvant, genetic immunization, functional screen, immunomodulator, Parapox, Orf virus

## Abstract

**Background:**

The success of new sequencing technologies and informatic methods for identifying genes has made establishing gene product function a critical rate limiting step in progressing the molecular sciences. We present a method to functionally mine genomes for useful activities *in vivo*, using an unusual property of a member of the poxvirus family to demonstrate this screening approach.

**Results:**

The genome of *Parapoxvirus ovis *(*Orf virus*) was sequenced, annotated, and then used to PCR-amplify its open-reading-frames. Employing a cloning-independent protocol, a viral expression-library was rapidly built and arrayed into sub-library pools. These were directly delivered into mice as expressible cassettes and assayed for an immune-modulating activity associated with parapoxvirus infection. The product of the B2L gene, a homolog of vaccinia F13L, was identified as the factor eliciting immune cell accumulation at sites of skin inoculation. Administration of purified B2 protein also elicited immune cell accumulation activity, and additionally was found to serve as an adjuvant for antigen-specific responses. Co-delivery of the B2L gene with an influenza gene-vaccine significantly improved protection in mice. Furthermore, delivery of the B2L expression construct, without antigen, non-specifically reduced tumor growth in murine models of cancer.

**Conclusion:**

A streamlined, functional approach to genome-wide screening of a biological activity *in vivo *is presented. Its application to screening in mice for an immune activity elicited by the pathogen genome of *Parapoxvirus ovis *yielded a novel immunomodulator. In this inverted discovery method, it was possible to identify the adjuvant responsible for a function of interest prior to a mechanistic study of the adjuvant. The non-specific immune activity of this modulator, B2, is similar to that associated with administration of inactivated particles to a host or to a live viral infection. Administration of B2 may provide the opportunity to significantly impact host immunity while being itself only weakly recognized. The functional genomics method used to pinpoint B2 within an ORFeome may be more broadly applicable to screening for other biological activities in an animal.

## Introduction

Bioinformatic analyses of microbial sequences have changed the way searches for vaccine antigens can be conducted; researchers can avoid pathogen isolation, culturing, and fractionation as a means of target identification. However these "reverse vaccinology" approaches [1], in which sequences are evaluated relative to their likelihood of encoding protective antigens, carry some limitations such as i) our poor understanding of the characteristics of a pathogen component that make it protective and ii) the inability to query the substantial proportion of any microbial genome that carries no previously described homologs or motifs [2]. Therefore, empirical approaches to rapidly determine the natural functions or non-natural applications of genome-encoded products are needed [3]. Toward this purpose, there has been some progress in using genome databases to recombinantly produce proteins in cell culture or test tubes for printing nanoscale quantities onto glass microscope slides [4]. These protein arrays can be used to perform rapid, parallel *in vitro *screens if a slide-compatible functional assay is available. Genome-wide screening in mammalian cells using RNA interference and expression libraries has also gained much popularity [5]. However, these platforms do not provide the opportunity to assay activities at the organism level, such as an animal's immune activity.

We previously described an approach to vaccine antigen discovery that, similar to reverse vaccinology, does not require pathogen culturing but in contrast does not rely on bioinformatic analyses nor require recombinant protein production. Instead, it is based on the delivery of each gene *in vivo *and its direct functional testing as a vaccine candidate in host animals. Using expression library immunization (ELI) [6], pathogen genomes can be searched for protective antigens in streamlined multiplexed animal challenge assays [7-9]. Here, this parallel-testing strategy is extended for use in searching for any measurable biological activity encoded by a gene *in vivo*. It uses genomic sequence information to prepare genes for rapid and unbiased isolation of biological effectors. As demonstration, we chose a pathogen with a novel immune activity of potential application for therapeutic immune-modulation.

The *Poxviridae *Family includes many well-known pathogens. They carry complex genomes that encode a variety of mechanisms for subverting the immune responses of infected hosts [10]. The globally prevalent *Parapoxvirus ovis *(ORFV) causes Orf, an acute and highly infectious disease largely limited to sheep and goats, but which can be also transmitted cutaneously to people handling infected animals [11]. Epithelial infection leads to the development of pustular skin lesions and then scabs [12]. Virus is shed from the scab and no systemic spread occurs. Despite lesion repair and an initial vigorous inflammatory response, the ORFV-specific host immune response is transient and ineffective in viral neutralization, perhaps due to its unique immune regulation and skin epithelial niche [13]. Consequently, re-infection of the same host is common [14,15]. Administration of whole inactivated virus (iORFV) similarly shows clinical effect [16] and fails to prevent subsequent infections, rendering a conventional vaccine design against Orf disease impractical. However, these inactivated preparations hold strong immune-stimulating properties including transient interferon-gamma (γ-IFN) induction followed by autoregulatory T helper 2 cell cytokine activity that is not antigen specific [15,17-19]. Inactivated virus provides short term, non-specific immune protection against a number of viruses [16,17], and is sold as Baypamun (Bayer, Inc.), or more recently as Zylexis (Pfizer AH, Inc.), to prevent disease in herds during short periods of close confinement and stress, such as during transport and regrouping [20].

Failure of a host to sustain specific immunity against ORFV infection may relate to the effect of the infection on the skin's epidermal Langerhans and dermal dendritic cells (DC). ORFV has the ability to manipulate these antigen presenting cells (APCs) cells, central to initiating both innate and adaptive immune responses [21,22]. Histologic examination of pock lesions demonstrates that the sites of infection become densely packed with DC within two to four days of virus exposure [23]. Since they appear to be recruited and detained rather than having migrated away as would be expected following antigen exposure, they serve as a barrier to systemic infection and cannot initiate an adaptive response because they do not gain access to T cells in the lymph nodes. Furthermore, when the skin lesions fully resolve the DC are concomitantly sloughed off [23]. These observations suggested that ORFV carries one or more factors that are able to recruit and/or retain DC at the skin. One or both of these immune-related activities may contribute to the unusual characteristics of ORFV infection. Notably, iORFV inoculation does not stimulate DC accumulation yet does initiate a strong inflammatory cytokine response and transient non-specific immunity. Isolation of the putative immune-cell modulating component(s) might provide useful research and biomedical applications for it.

To mine the ORFV genome empirically for the immune-cell regulator gene(s) that cause the accumulation of DC-like cells at a site of skin inoculation, the genomic sequence of the ORFV strain used in the Baypamun and Zylexis products, D1701, was determined. This complete yet low coverage sequence information was used to specify oligonucleotide (oligo) primers for the amplification of all predicted open reading frames (ORFs). This ORFeome was placed in linear expression elements (LEEs) [24] and delivered into mice to directly assay for the desired innate immune activity. The B2L gene was identified as eliciting DC-like cell accumulation and also other immune-modulating activities. Regulation of DC-like cells might be generally useful in vaccination or other immunomodulation strategies [25]. This approach of screening a library directing in an animal for a biological activity may have further utility.

## Results

### Sequence determination

The D1701 isolate of *Parapoxvirus ovis *(ORFV) was used to prepare the immune-stimulating product Baypamun; therefore, genomic (g)DNA from this virus was obtained (gift from Tobias Schlapp, Bayer). The viral gDNA was shotgun cloned for sequencing. The final assembly consisted of 134,040 nucleotides and is reported [**Genbank: **HM133903]. Three complete ORFV (Orf) genomes are available in the NCBI database (OV-NZ-2, OV-IA82, and OV-SA00). The NZ-2 strain is most closely related to D1701, with > 95% nucleotide sequence identity. The majority of the differences between the sequences of D1701 and NZ-2 are found toward the flanking terminal repeat regions. An electronic MacVector Genescan course analysis of the assembled D1701 genome using a minimum ORF encoding length of 90 aa yielded 288 putative proteins. The annotated ORFV genomes in NCBI each have only 134 identified ORFs, indicating that our parameters are relaxed. We chose not to modify these determinations so as to be most inclusive and use the functional screen to eliminate non-ORFs. Sequence comparison (BLAST) of the D1701 strain to the NZ-2 ORFV strain identified three additional putative ORFs; these were added to the list to obtain a total of 291 sequences to explore. We determined that D1701 B2L was not recognized in our annotation because of a single base sequencing error that created a predicted frameshift at amino acid position 126 and a stop codon at aa position 299. Whereas this led to the design of a shortened ORF that appeared to encode a divergent aa sequence, the NZ-2 sequence comparison enabled us to identify the sequencing error and retrieve the wild type full length ORF. The B3L gene homolog had not been designated as an ORF because the translated product contains only 89 amino acids and our algorithm required a minimum of 90 amino acids; the B3L gene [26] was included in the screen.

### Functional screening of an ORFV library for immune cell recruitment activity

**Figure 1:**
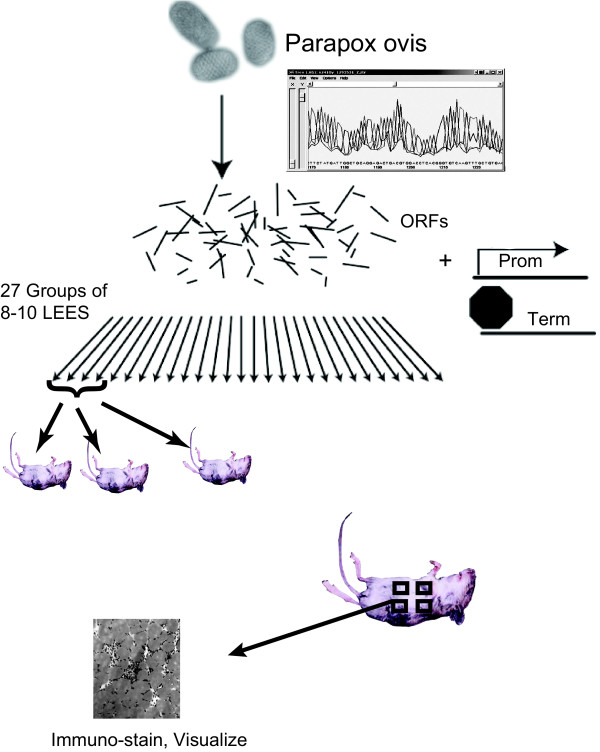
**An *in vivo *strategy for functionally searching the *parapoxvirus ovis *genome for useful ORFs**. The genomic sequence of *parapoxvirus ovis *(ORFV) strain D1701 was determined, and ORFs were identified. Sequence results were used to design PCR primers for gene amplification. The library of parapoxvirus ORFs (ORFeome) was generated and then partitioned into 27 pools of 8 to 10 ORFs each. Mammalian promoter and terminator expression elements were added to each pool, treated to expose single stranded end sequences, and then assembled into LEEs by sequence specific annealing. The ORF-expressing LEE pools were precipitated onto gold microparticles and biolistically delivered into the abdomen skin of BALB/c mice. To limit the number of animals used, each mouse was gene-gun inoculated with four to six different LEE pools at distinct abdominal sites. Each pool was tested in triplicate, in 3 different mice. Tissue sections were harvested 4 days later, cryo-sliced and immuno-probed to identify MHC II expression (I-A^d^/I-E^d^) and visualize cell morphology.

The viral ORFeome screening strategy is illustrated in Figure 1. Based on the sequence annotation, 257 parapoxvirus ORFs were successfully PCR-amplified and these were randomly distributed into 27 pools of 8 to 10 ORFs each. All ORF pools were non-covalently linked to a mammalian promoter and termination element to obtain LEEs as previously described [24]. Since ORFV does not infect rodents, mice cannot serve as a model of host infection; however, they can serve as hosts for exploration of ORFV-elicited immune activity. The 27 pools were biolistically delivered into separated skin sites on the abdomen of BALB/c mice. Each mouse was inoculated by gene gun (shot) with 4 to 6 pools; each pool was delivered to 3 different mice. Four days after inoculation, the mice were sacrificed and the shot sites were harvested. Tissue sections were screened for DC-like cell accumulation by immunostaining cryo-slices for major histocompatibility (MHC) class II positive cells (anti-I-A^d^/I-E^d ^antigen reactivity). While multiple cell types in addition to dermal and epidermal DC have been identified within the skin that stain positively for MHCII I-A/I-E expression (namely macrophages, endothelial cells, and some T cell subclasses [27,28]), the positively staining cells appeared with DC-like cell morphology. Therefore, any newly observed I-A^+^/I-E^+ ^cell staining is expected to include the migratory DC, although other resident, non-migratory immune cell types will also contribute. A skin site inoculated with a LEE-encoding luciferase (LUC) was used both to normalize gene delivery efficiency and to serve as an irrelevant (non-ORFV) gene-inoculated tissue section. Viral infection could not serve as a positive control since mice are not hosts, and the inactivated virus preparation has been shown by us and others not to stimulate immune cell accumulation.

**Figure 2:**
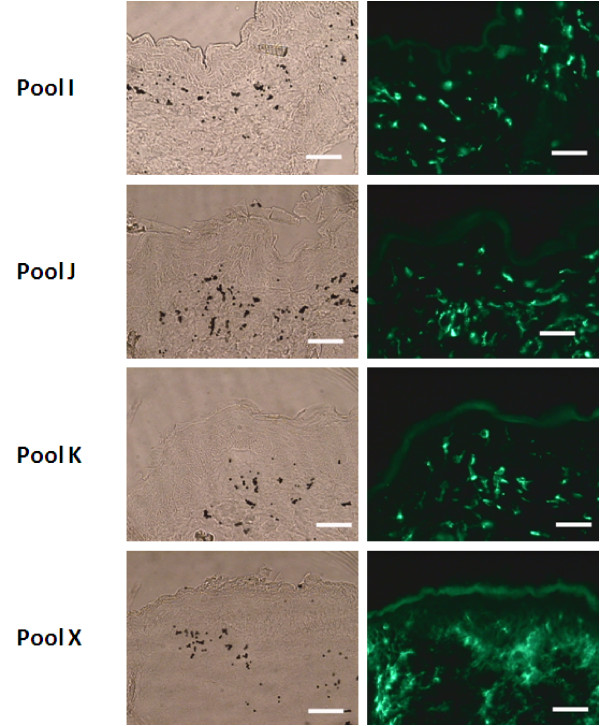
**Screening an ORFV expression library for genes encoding products that direct accumulation of MHCII-positive cells. Left panels**. Light microscope view pool-inoculated tissue sections, showing the distribution and density of gold microparticles delivered to cells by gene gun. **Right panels**. Tissue sections prepared from the same pool-inoculated mouse skin shown in left panel were stained with FITC-labeled antibody 2G9 (anti- I-A^d^/I-E^d^) to visualize I-A^+^/I-E^+ ^immune cells. Each panel shows skin samples obtained 4 days after gene gun inoculation with pools of mixed-ORFs (2 μg of total pool DNA per site). Tissue staining densities within sections harvested from mice inoculated with pools I, J, and K are representative of all but X. The sections obtained from the mice shot with pool X displayed an increase in staining. Scale bars are 200 micron.

**Figure 3:**
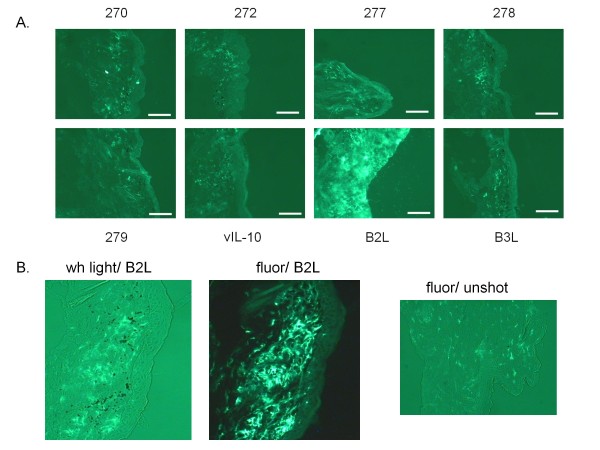
**Testing of individual ORFs comprising parapoxvirus library pool X identifies the B2L gene**. The ORF-expressing LEEs from pool X were inoculated individually into mouse abdomen sites using the gene gun (1 μg LEE per site), in triplicate, and skin sections were obtained 4 days later. Samples were processed and immuno-stained for the MHC II surface marker I-A^d^/I-E^d ^with antibody 2G9. Scale bars are 200 micron. **A. **Immunostains of tissue sections from ORFV ORF-expressing LEEs comprising pool X. Viral gene numbers are indicated alongside corresponding skin sections. Each of the eight representative tissue sections is presented as an overlay of FITC labeled anti-I-A^d^/I-E^d ^fluorescent microscopy. **B. **The same section of skin inoculated with B2L was viewed under both white visible (wh) light to observe gold particles **(left panel) **and under fluorescence (fluor) to visualize FITC-labeled class II positive cells (I-A/I-E+) (**middle panel**). An untreated section of skin was stained with FITC labeled anti-I-A/I-E and visualized under fluorescence (fluor) (**right panel)**.

The densities and morphologies of anti-I-A/I-E-visualized MHC class II-positive cells were similar among the bombarded tissue samples save for those shot with ORF-pool X. This is displayed in representative samples shot with pools I, J, and K as compared to pool X (Figure 2, right column). The pool X-shot tissue sections exhibited a more diffuse and higher level of staining relative to sites inoculated with any of the other 26 pools, an irrelevant luciferase (LUC) LEE, or even unshot skin (Figure 3b, right panel and unpublished results). Thus, pool X appeared to contain a gene whose product modified the accumulation and/or distribution of MHC II+ cells. The same four tissue sections are shown under white light microscopy to visualize microparticles so as to compare delivery efficiencies among pool tests (Figure 2, left column). Comparison of left and right columns can also be used to assess positions of gold particles relative to positions of I-A^+^/I-E^+ ^cells.

Pool X was comprised of eight ORFs; this included the three ORFs identified by comparison to the NZ-2 strain sequence (B2L, B3L and vIL10). The remaining five were predicted from the D1701 sequencing results (270, 272, 277, 278, and 279). The NZ-2 B2L gene product had been previously identified as the homolog of the vaccinia (VACV) virus major envelope antigen gene, F13L [26,29]. The truncated D1701 B2L ORF described above resided in pool J, which did not induce the accumulation of I-A^+^/I-E^+ ^cells (Figure 2a). Pool J was retested multiple times in multiple mice to confirm this negative result (data not shown).

The effect of each of the ORFs comprising pool X on immune cell accumulation was tested by delivering them separately (1 μg/ORF) at distinct sites on the abdomen of mice, in triplicate. The original X pool and a LEE expressing the LUC ORF were retested as positive and negative controls, respectively (data not shown). The tissue sections removed from each inoculation site were stained for anti-I-A/I-E antibody reactivity (Figure 3a). Skin sections inoculated with the ORF encoding B2 displayed increased densities of MHC class II positive cells relative to control tissues (Figure 3a). In a confirmatory experiment, both white light and fluorescent images of the same B2L-shot tissue section were visualized so that immune cell localization and gun microparticle distribution could be compared (Figure 3b, left and middle panels). As indicated by the fluorescent image in the middle panel, inoculation of abdominal skin with the full length B2L ORF- D1701 led to DC-like cell accumulation relative to unshot tissue (Figure 3b, right panel).

### B2 protein displays immune cell accumulating and adjuvant-like activities

**Figure 4:**
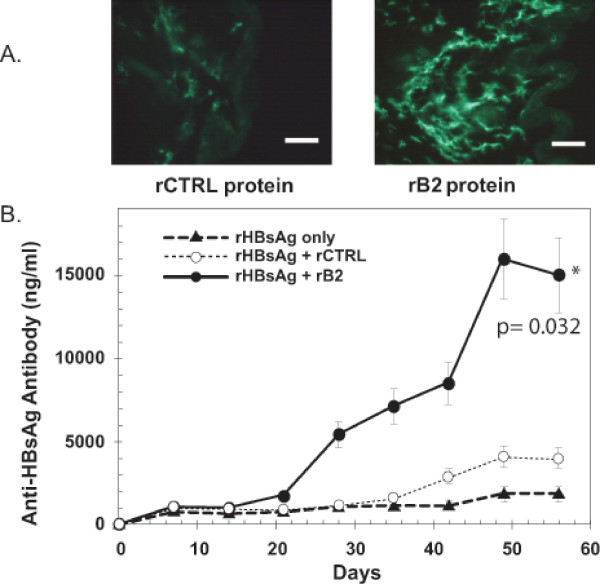
**The B2 protein directs accumulation of immune cells and enhances an antigen-specific antibody response. A**. Mice were injected i.d. in the abdomen with 50 ng of the irrelevant recombinant (r)SRB4 protein (rCTRL) (**left panel**) or recombinant (r)B2 protein (**right panel**); the 2 proteins were injected into separate abdominal sites of 3 mice. Both proteins were produced in *E. coli *by the same protocol and identically purified. After 4 days, immune cell densities were measured in harvested skin by 2G9 immuno-staining, as described in Figures 2 and 3. Scale bars are 40 micron. **B**. Mice were co-injected i.d. in the abdomen with 1 μg rHBsAg alone (▲), 1 μg rHBsAg with 50 ng of rB2 protein (●), or 1 μg rHBsAg with 50 ng of the rSRB4 control protein (rCTRL) (O). Sera collected over a 2 month time-course were assayed for antibodies to HBsAg by ELISA, with an HRP-readout as described in Material and Methods. No boosts were administered. Antibody levels of experimental samples were calculated relative to absorbance (A_450_) values measured for known amounts of a commercial anti-HBsAg antibody (Seradyn, Inc). Calculated antibody levels are plotted; error bars are SDs from the arithmetic mean. Student's t-test was used to compare antibody levels at day 56.

The D1701 B2 protein was recombinantly expressed and affinity-tag purified. A yeast protein, SRB4, was produced in the same *E. coli *expression vector and purified in the same manner in parallel to serve as an irrelevant protein control. When intradermally (i.d.) injected into mouse abdominal skin, recombinant (r)B2 protein led to the accumulation of DC at the site of inoculation with efficiency similar to that seen following genetic inoculation (Figure 4a and 3). Neither an irrelevant recombinant protein (Figure 4a) nor the buffer control (data not shown) increased the local density of DC-like cells. For comparison, rB2 from the ORFV isolate NZ-2 was also produced and purified. Injections of this protein also elicited DC-like accumulation at the skin site (Additional file 1).

In an immunization experiment, mice were i.d. injected with i) rHBsAg protein only, or co-injected with ii) rHbSAg + rSRB4 (control), or iii) rHbSAg + rB2. Administration of rHBsAg by itself or with the rSRB4 protein control (rCTRL) did not stimulate a significant anti-HBsAg antibody response. However, co-immunization with rB2 stimulated a strong antibody response (Figure 4b). For example at day 56, rHBsAg alone generated titers significantly lower than that generated by rHBsAg + rB2, *p *= 0.029. Similarly, rHBsAg + rCTRL generated titers significantly lower than that generated by rHBsAg + rB2, *p *= 0.032. To explore the possibility of an antibody response to the adjuvant itself, these same sera were also tested in an ELISA against rB2 as antigen. No reactivity to B2 was detected by ELISA (Additional file 1) or immunoblot, when it had been administered to the mice in either protein-format (Additional file 2) or gene-format (data not shown).

### B2L as modulates infectious and noninfectious disease. models

**Figure 5:**
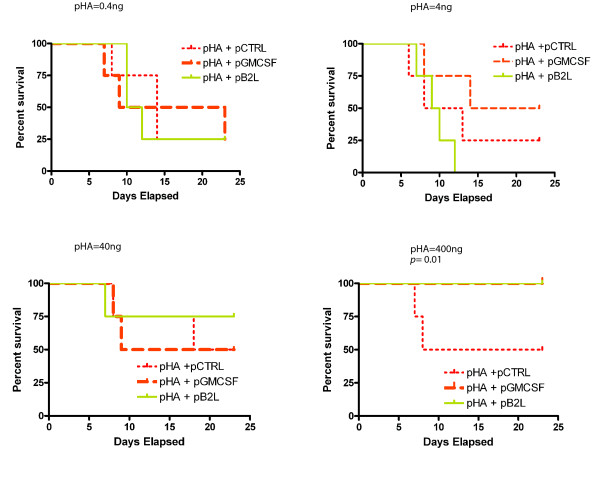
**Protective immunity against influenza virus conferred by a hemagglutinin gene vaccine is enhanced by co-administration of the B2L gene**. Groups of BALB/c mice were immunized with a series of pCMVi-HA (pHA) doses: 0.04 ng, 4 ng, 40 ng, and 400 ng, as indicated above each graph, and represent total mouse doses which were delivered in 2 gene gun discharges (shots). This gene vaccine plasmid (pHA) was co-delivered with 1 μg pUC119 as a negative control plasmid (pCTRL) (short dashes), 1 μg pCMVi-B2L (pB2L) (solid line), or 1 μg pCMVi-GMCSF (pGMCSF) as a positive control plasmid (long dashes), delivered in 2 shots. Animals were given a second immunization with the same inocula and dose 3 weeks post-prime. Mice were challenged with a lethal intranasal dose of influenza A virus 6 weeks after the boost. All mice in the control, unimmunized group died on or before day 12. The Kaplan-Meir survival curves for each group of mice (8 mice per group) are displayed. A parametric survival test (Prism 4, Graphpad, Inc.) was used to measure significant differences between survival rates observed at the 400 ng HA gene vaccine dose.

The ultimate goal of an immunization is to provide protection against disease. Therefore, the effect of co-delivering pCMVi-B2L as an adjuvant for an influenza gene vaccine was evaluated in a viral protection assay. Mice were administered a construct expressing the protective hemagglutinin (HA) gene (pCMVi-HA) in a dosage series of 0.4 ng, 4 ng, 40 ng, and 400 ng total mouse dose along with a 1 μg total mouse dose of either pCMVi-B2L, pCMVi-mGMCSF, or a control plasmid (pUC119). The same inocula were administered three weeks post-prime; at six weeks post-boost all animals were challenged with a lethal dose of influenza A virus and survival was monitored daily. When the vaccine was delivered with only the control plasmid, even the highest dose of pCMVi-HA did not protect 50% of the mice from viral challenge. By contrast mice co-immunized with pCMVi-HA and a plasmid expressing either i) B2L or ii) the established genetic-adjuvant GMCSF were fully protected. This vaccine enhancing effect was antigen dose dependent. At the intermediate pCMVi-HA doses, co-delivery of either the murine GMCSF gene (4 ng) or B2L gene (40 ng) conferred a protective trend relative to the control vaccine. At the lowest vaccine dose (0.4 ng), only 20% of the animals survived in all three groups indicating that both the B2L and positive control adjuvant expressing plasmids were unable to enhance the antigen-directed protection at this dose. As displayed in Figure 5, a log-rank survival analyses of the Kaplan-Meier curves indicates a significant difference between the adjuvanted and unadjuvanted gene vaccine at 400 ng (*p *= 0.01).

**Figure 6:**
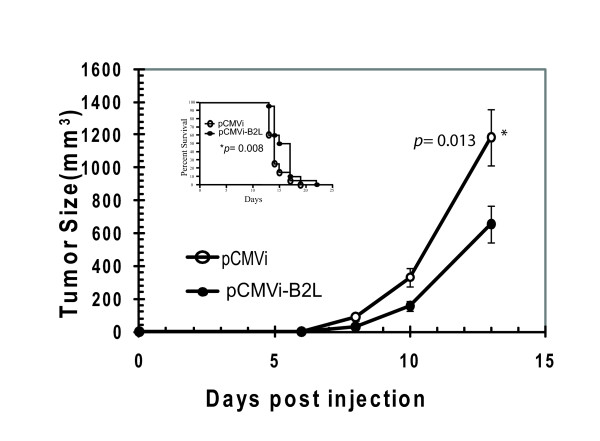
**Immunization with the B2L gene reduces tumor growth in a murine model of aggressive melanoma**. Two groups of 20 C57BL/6 mice were injected s.c. with 5 × 10^4 ^B16-F10 cells. Twenty-four hours later, the test group (●) was biolistically administered pCMVi-B2L to both sides of both ear pinnae (4 shots of 0.5 μg totaling 2 μg per mouse). The control group (O) was administered the same dose of empty vector (pCMVi). Animals were monitored daily; on day 6, tumors were palpable and these were measured in triplicate by two individuals. Tumor growth was similarly measured and recorded on days 8, 10, and 13. Average tumor sizes within each group are plotted; error bars represent SDs from the arithmetic mean. Survival curves for the same mice are displayed as an inset with a *p *value derived to statistically compare control and B2L-immunized groups (Prism4 software, Graphpad, Inc.).

In addition to infectious diseases, vaccines can be envisioned to defend against non-infectious diseases such as cancer; however, common tumor-specific antigens are difficult to identify. Using a murine transplantable model of an aggressive melanoma cancer, we tested the possible effect of pCMVi-B2L, without a co-delivered antigen, as a non-specific defense against tumor development. Two groups of 20 C57BL/6 mice were injected subcutaneously (s.c.) with the B16 sub-line B16-F10. The following day, the test group was genetically immunized with the mammalian expression plasmid pCMVi-B2L and the control group received the empty vector (pCMVi). Palpable tumors were detected at day 6 post cell injections; tumor dimensions were measured at days 8, 10, and 13. Calculated volumes plotted in Figure 6 show a significant reduction in the tumor sizes in the pCMVi-B2L immunized mice relative to vector only controls (*p *= 0.013). Sacrificed animals were recorded through day 25, establishing a significant difference in survival curves (*p *= 0.008). Immunization of BALB/c mice with a similar B2L expression plasmid (pPCR3.1-B2L) led to a modest but measureable reduction in tumor sizes 20 days following injection of the breast cancer cell line 4T1 (*p *= 0.036) (data not shown).

## Discussion

New genomic sequence information from a pathogen was rapidly converted into gene function data using a direct screen for biological activities of interest. This was accomplished by taking advantage of linear gene expression, a multiplexed pooling strategy, and genetic immunization. In this example screen, a genome was queried for an immunomodulating activity; however, a similar strategy could be applied to query any genome for any gene-encoding activity for which one can design a bio-assay. The low coverage sequencing reads performed in this work were sufficient to identify all ORFs and design primers for PCR. Higher coverage reads would afford the opportunity to synthetically build genomes, and thereby further eliminate the need for culturing pathogens even if only for genomic DNA template production. As time and cost of both large scale sequencing and gene building continue to decrease, this latter approach is increasingly attractive.

A *parapoxvirus ovis *gene responsible for the manipulation of host immunity was initially identified in days, using less than 30 mice. In particular, expression of the ORFV B2L gene was found to elicit the accumulation of DC-like cells at the site of skin inoculation. We did not establish whether this accumulation is a result of immune cell retention or recruitment. Dermal and epidermal (Langerhans) DC are distributed in lattice-like patterns within the skin [30,31], comprising an estimated 10% of the dermal cells [27]. This lattice is observed when untreated mouse skin is immuno-stained for MHC II-expressing cells. When skin is bombarded with gold microparticles, many of the DC disappear from the shot skin site [32,33]. These cells react to bombardment by migrating to the draining lymph node where they can activate T cells and other immune cells [34,35]. Microparticle densities visualized under white light appeared similar among the many tissue sections bombarded with particles carrying a pool of LEEs. However, migrating DC represent only a portion of I-A^+^/I-E^+ ^skin cells, therefore their loss, or lack of loss, may not be measureable. More informatively, overlaying of the white light and fluorescent views of the shot tissue sections indicate that skin samples shot with either pool X or the individual B2L gene display significant areas of fluorescence that do not correlate with the position of a particle. Whereas, samples shot with other pooled or individual constructs displayed a close correspondence between particles and fluorescence (Figure 2 and 3). These results indicate that non-resident I-A^+^/I-E^+ ^immune cells were recruited to the shot site after bombardment with B2L. Non-resident DC have been previously shown to be recruited to a skin site upon ORFV infection [23].

Relative to other resident skin cell types, MHC II-positive DC are the most potent initiators of an immune response [33]. However, the increased density of DC at an immunization site could influence the generation of a subsequent adaptive response with several possible outcomes. For instance, by causing resident DC to be retained and others further recruited to the skin rather than homing to the draining lymph node, an immune response might be diminished. Alternatively, maintaining the DC at the immunization site may provide additional opportunity to process antigens synthesized by neighboring cells such as keratinocytes before eventually migrating to the lymph node, thus initiating a stronger immune response. Finally, B2L co-delivery with an antigen may have no effect on the antigen specific response.

We showed that co-delivery of B2 protein with an antigen (HBsAg) elicited enhanced levels of antigen-specific antibodies (Figure 4). Protective immunity elicited by an influenza gene vaccine was also enhanced by co-administration of the B2L gene (Figure 5). In a gene vaccine dose titration, a positive trend for adjuvanted protection was observed at intermediate vaccine doses but not the lowest dose, indicating protection was antigen specific and dose dependent. We additionally found that gene gun administration of the B2L expression construct to mice, without an antigen expressing construct, was able to reduce the rate of tumor growth in a transplantable model of cancer (Figure 6). The non-specific protection provided by B2L is reminiscent of the short-term protection conferred by either live ORFV or iORFV against viral infections; however, iORFV does not also cause I-A^+^/I-E^+ ^cell accumulation, as do B2L and live virus. This seems to suggest that delivery of B2L is immunologically more similar to live infection than delivery of whole inactivated virions.

Delivery of the B2L expression construct arguably provides three possible effector molecules for the observed immune activities: DNA, RNA, and protein. However, delivery of the DNA construct carrying a full length B2L gene but expressing a truncated B2 protein did not hold modulating activity, whereas direct delivery of purified full length B2 protein did (Figure 4, Additional file 1). These results indicate that the B2 protein is the relevant effector molecule, and that the effector activity requires the C-terminal portion of the protein.

Neither the mechanism of B2-induced accumulation of immune cells nor its mechanism of enhancing specific or non-specific responses is clear. However, informatics analyses are suggestive. B2 is the homolog of vaccinia virus (VACV) F13, also called p37 [26], which carries a number of known features. This palmitoylated protein is the major antigen on the surface of enveloped virions, and is required for viral envelopment and egress [36]. F13 is one of seven virus-encoded envelope proteins which is present in the intracellular enveloped virus (IEV), cell-associated enveloped virus (CEV), and extracellular enveloped virus (EEV) [37]. Two others of these seven VACV envelope proteins, B5 and A33, are components of the "4-pox" subunit vaccine candidate for smallpox [38]. A33 targets F13 to the IEV, and these two proteins immunoprecipitate as an A33/F13 complex from infected cell lysates [39]. Proteins F13 and B5 co-localize forming a disulfide bonded complex that is required for efficient and complete wrapping of the intracellular mature virus [29,37,39].

ORFV is unique relative to VACV and other orthopoxviruses in its lack of any B5 homolog. Perhaps the lack of an homologous B5/F13 complex is relevant to the unique immune cell accumulation activities of ORFV. By contrast, an A33 homolog does exist in ORFV. Stage-dependent trafficking of B2 by an A33 homolog during the ORFV infection cycle might be relevant to the immune cell accumulating activity of live-virus and purified B2, which iORFV preparations such as Baypamun, lack. Inactivated virions represent one snapshot of surface protein landscapes, whereas a live virus could cycle proteins to and from the surface.

Vaccinia F13 carries a conserved HKD-like motif that is found in members of the phospholipase D (PLD) gene superfamily, which encodes the catalytic site for phospholipase activity or simply a lipid binding site [40]. Lipids play a regulatory role within the immune system [41]. Mammalian PLDs have been well studied because of their role in membrane vesicular trafficking and critical signal transduction cascades [37]. Mutation of the HKD-like motif in F13 of VACV leads to loss of cell to cell viral spreading, indicating that it is an active superfamily member. It appears to encode a lipid binding rather than modifying activity [40], such as do toll-like receptors. Notably, this C-terminally-located PLD domain is found at aa positions 318-342 of B2. In our experiments, the initially designed B2L ORF did not display immune I-A^+^/I-E^+ ^cell accumulation activity in our expression screen. This truncated ORF was amplified from D1701 gDNA, therefore it carried the correct sequence, not the sequencing error at aa position 126. However, the prematurely positioned 3' primer caused a truncation at position 299 (because the 3' primers of the library were all designed with in-frame stop codons).

B2L of ORFV strains D1701 and NZ2 differ at the nucleotide level at only 32 of 1134 nucleotides and at the protein level there are only 9 aa substitutions. Table 1 displays the protein sequences encoded by the two B2L genes. The C terminus is invariant; there are no amino acid changes in the PLD-related region of the protein. By contrast there are several aa changes relative to the C terminus of VACV F13, as highlighted in Table 2. These comparisons are consistent with a distinction between activities of the VACV versus ORFV homologs. While VACV infection does not lead to immune cell accumulation, we did not evaluate the activity of VACV F13 in isolation; therefore, we cannot ascertain whether or not it independently carries B2-like modulatory activity.

**Table 1:** Sequence comparison of the B2 protein of ORFV strains D1701 and NZ2^1,2^.

D1701_B2	MWPFSSIP**V**G A**Q**CRV**L**ETLP AEVASLAQGN MSTLDCFTAI AESAKKFLYI CSFCCNLSST
NZ2_B2	MWPFSSIP**L**G A**D**CRV**V**ETLP AEVASLAQGN MSTLDCFTAI AESAKKFLYI CSFCCNLSST
**D1701_B2**	KEGVDVKDKL CTLAKEGVDV TLLVDVQSKD KDADELR**A**AG VNYYKVKVST **R**EGVGNLLGS
NZ2_B2	KEGVDVKDKL CTLAKEGVDV TLLVDVQSKD KDADELR**E**AG VNYYKVKVST **K**EGVGNLLGS

**D1701_B2**	FWLSDAGHWY VGSASLTGGS VSTIKNLGLY STNKHLAWDL MNRYNTFYSM IVEPKVPFTR
NZ2_B2	FWLSDAGHWY VGSASLTGGS VSTIKNLGLY STNKHLAWDL MNRYNTFYSM IVEPKVPFTR

**D1701_B2**	LCCA**V**VTPTA TNFHL**N**HSGG GVFFSDSPER FLGFYRTLDE DLVLHRIENA KNSIDLSLLS
NZ2_B2	LCCA**I**VTPTA TNFHL**D**HSGG GVFFSDSPER FLGFYRTLDE DLVLHRIENA KNSIDLSLLS

**D1701_B2**	MVPVIKHA**G**A VEYWP**R**IIDA LLRAAINRGV RVRVIITEWK NADPLSVSAA RSLDDFGVGS
NZ2_B2	MVPVIKHA**S**A VEYWP**Q**IIDA LLRAAINRGV RVRVIITEWK NADPLSVSAA RSLDDFGVGS

**D1701_B2**	VDMSVRKFVV PGRDDAAN***NT KLLIVDD***TFA ***H***L***TVAN***L***DGT HY***RYHAFVSV NAEKGDIVKD
NZ2_B2	VDMSVRKFVV PGRDDAAN***NT KLLIVDD***TFA ***H***L***TVAN***L***DGT HY***RYHAFVSV NAEKGDIVKD

**D1701_B2**	LSAVFERDWR SEFCKPIN
NZ2_B2	LSAVFERDWR SEFCKPIN

^1^Amino acid differences are underlined.

^2^Key residues of PLD domain are italicized.

**Table 2:** Sequence of the highly conserved region IV of phospholipase D1 family members found in parapox and vaccinia viruses.

PLD1 family member	PLD1 domain amino acid sequence
**ORFV B2**	NT *KLLIVDDTFA *HLTVANLDGT HY

**VACV F13^1^**	N*N *TKLLIVDDEY* VHITSANFDGT HY

^1^Amino acid differences in the VACV domain sequence relative to the ORFV sequence are underlined

Further work will explore whether B2 is responsible for any other activities related to the immune system. For example, B2 may stimulate the uptake of exogenous antigen or stimulate chemokine activity. As a read-out of an adaptive response, we have used antibody reactivities. Since B2L administration enhanced the effectiveness of a viral gene vaccine it may also influence cellular mediated immune activities. The effect of B2L expression on tumor development in the absence of antigen suggests a non-specific immunity is elicited against a chronic disease. Testing B2L administration prior to an infectious challenge will determine whether this molecule alone can also protect against an acute disease.

## Conclusions

A novel immunomodulator, B2, was isolated from an Orf virus in a functional screen of its ORFeome. B2 was identified by its effective accumulation of immune cells at a site of inoculation. However several practically significant activities were also established. B2 performs as an enhancer of antigen specific immunity, it acts as a non-specific immune enhancer in the absence of an antigen, and it is itself non-immunogenic.

Whereas mechanistic studies are typically performed as a means to the identification of a factor responsible for a given activity, the functional genome mining approach presented here enabled the factor to be identified and isolated before its mechanism of action was explored. Mechanistic studies can now be conducted with better molecular tools. Characterizing B2 and its activities should facilitate our ability to modulate and control immune responses. Recent trends in vaccine development are aimed at identifying subunit vaccines rather than producing the conventional whole-pathogen based ones. This creates a greater need for developing a toolbox of safe adjuvants to compensate for the reduced immunogenicity of the pure component formulations.

## Methods

### Reagents

Purified parapoxvirus ovis DNA (D1701 strain) was obtained from Tobias Schlapp of Bayer Pharmaceuticals, Germany. Female BALB/c and A/J mice were purchased from Harlan Laboratories (Indianapolis, IN). Oligo primers for PCR amplification of individual ORFs were synthesized in house using either an ABI synthesizer or the MerMade3 high throughput synthesizer [42]. The ORFV-specific primers were designed with a common 15 nucleotide sequence at the 5'ends carrying 5 deoxy-uracil (dU) bases. Distinct dU-containing sequences were designed for the forward and reverse primer sets. After PCR amplification, the dU stretches can be removed by treatment with uracil-DNA glycosylase (UDG), exposing defined 3' overhanging sequences. These single-stranded regions are designed to hybridize to complementary overhangs, similarly generated, on PCR products carrying a CMV promoter and hGH terminator [43] FITC-conjugated rat anti-I-A^d^/I-E^d ^antibody (2G9), rat anti-I-A^k ^antibody (11-5.2) and unlabelled rat anti-CD16/CD32 (Fc block, 2.4G2) were purchased from Pharmingen (San Diego, CA). Rat IgG was purchased from Jackson Laboratories (West Grove, PA). Tissue Freezing Medium (Triangle Biomedical Sciences, Durham, NC) and Superfrost Plus microscope slides were obtained from Fisher Chemical Co (Norcross, GA). Vectashield fluorescence microscopy mounting medium was obtained from Vector Laboratories (Burlingame, CA).

### Sequencing of ORFV D1701

The D1701 viral genomic (g)DNA was digested with BamHI and BglII then cloned into the pUC118 vector [44]. Random clones from this library were sequenced from both ends. Sequencing reads were assembled and gaps filled by walking into library clones with primers generated against newly determined sequence or by PCR amplification from intact ORFV genomic DNA. Sequence coverage varied from a minimum of 3 to over 50 reads per segment, which would be considered a relatively low resolution determination. The final assembly consisted of 134,040 nucleotides with 32 ambiguous positions. The terminal repeat sequences at the ends of the viral genome appear to be incomplete in the random library. These were not resolved because in all three currently sequenced Orf viruses there is at least 2400 bp from the reported ends to the first or last protein coding segment.

### Production of ORFV genes and expression element components

The sequence of the 134 kb ORFV genome was searched for ORFs using the MacVector Genescan sequence analysis program. The parameters were set such that each ORF required a start and stop codon and encoded a protein with a minimum length of 90 amino acids (aa). The search included both strands, in all possible reading frames and did not exclude overlapping reading frames. This search yielded 288 possible ORFs. In addition, three full-length protein-coding sequences from the NZ-2 strain of ORFV virus were present in the NCBI sequence database that were similar to D1701 sequences that we had not annotated as ORFs. We prepared primers based on the NZ-2 sequences and amplified these regions of D1701 for inclusion in the library. ORFV-specific primer sets were designed and synthesized for all D1701 ORFs.

PCR products were obtained using the following conditions. Each 50 μl reaction contained 10 pmol of forward and reverse primer, 5 ng ORFV genomic DNA template, 10 mM Tris-HCl, pH 8.8, 50 mM potassium glutamate, 2 mM MgCl_2_, 0.2 mM dNTP, 1 M betaine and 2.5 U Taq polymerase. Each reaction was overlaid with mineral oil and incubated according to the following parameters in a Strategene Robocycler. An initial cycle consisted of 3 min denaturation at 95°C, 1 min annealing at 55°C and 1 min extension at 72°C. This was followed by 10 cycles of 1 min denaturation at 94°C, 1 min annealing at 55°C and 1 min extension at 72°C and 24 cycles of 1 min at 94°C, 1 min at 50°C and 1 min at 72°C. The PCR was finished with a 5 min extension at 72°C. All PCR products were assessed by electrophoresis in 1.2% agarose gels and DNA concentration was determined using a Hoeffer fluorometer and Hoechst dye and plasmid DNA as standard.

PCR amplification of each ORF from the genomic template yielded 257 specific products of the correct lengths and high yields. Amplification reaction products for the remaining ORFs did not meet these criteria so were not included in the library. The untested ORFs represent 12% of our annotated list and a similar proportion of the ORFV ORF homologs in the NCBI database (26 of 134). The library of 257 ORFV ORFs was randomly partitioned into 27 pools, each comprised of 8 to 10 ORFs. Batches of CMV promoter and hGH terminator fragments were generated by PCR with dU-carrying primers, as described for the ORFs. The downstream end sequence of the promoter element and the upstream end sequence of the terminator sequence were designed such that Uracil DNA-glycosylase (UDG) treatment would expose 3' single stranded stretches complementary to a 3' overhang on the corresponding ORF ends.

### Preparation of ORFV LEE library

The quantitated and quality-confirmed ORF PCR products were pooled (400 ng of each) into groups of 8 to 10 and then purified using Qiaquick spin columns per supplied protocols (Qiagen, Inc., Valencia, CA). The purified DNA pools were eluted from the columns in 50 μl 10 mM Tris-HCl, pH 8.5. The CMV promoter and the hGH terminator elements were combined at an equal molar ratio with each of the 27 ORF pools (1 μg). UDG (O.5 unit) was added in a final volume of 50 μl 1X Buffer C (Promega Corp., Madison, WI). The samples were incubated at 37°C for at least 30 min, heated to 75°C for 15 min and mixed with an equal volume of 1 M KCl. After incubating an additional 15 min at 72°C, the components were allowed to anneal by slowly cooling to room temperature. The assembled LEEs were precipitated by the addition of 1/10 volume of sodium acetate and 2 volumes of ethanol. The precipitated DNA was collected by centrifugation and washed with 70% ethanol and allowed to dry before it was resuspended in water and loaded on gold for biolistic inoculation into abdominal skin [24].

### Gene gun inoculation and skin sampling for library testing

The desired amount of total DNA was precipitated onto gold beads using a calcium-spermidine protocol previously described [6]. The DNA-gold complexes were then spotted onto kapton membranes and dried (2 μg total DNA per kapton). The complexes were loaded into cartridges and delivered into mouse abdominal skin that had been prepared by treatment with shears and Nair depilatory using a custom hand held gene gun (Rumsey-Loomis, Ithaca, NY). Each BALB/c mouse was inoculated (shot) with DNA-gold complexes at 4 to 6 distinct abdominal skin locations, and each shot carried a different LEE pool. Each inoculum was delivered into 3 different mice to serve as biological replicates for each pool test. The sites of inoculation were evaluated 4 days later. Mice were euthanized and the inoculation sites were isolated; visible gold particles facilitated relocation of shot sites. Skin segments were trimmed of surrounding tissue and then immersed in cryoprotective medium and quickly frozen in liquid nitrogen. Thin-sections (8 μm) were cut in a Leitz cryostat, picked up on Superfrost plus microscope slides and stored at -20°C for subsequent fixation and staining.

Frozen sections were equilibrated to room temperature for 10 min then fixed by immersion in cold acetone for 10 min. The fixed sections were washed 3 times in phosphate buffered saline pH 7.3 (PBS). Non-specific adherence of rat IgG was blocked by treatment of the sections with a cocktail of Fc block reagent and rat IgG in PBS. After a 30-min incubation at room temperature, this blocking solution was replaced by FITC-conjugated rat anti-I-A^d^/I-E^d ^antibody (2G9) and incubation continued for 2 hours. The sections were washed 3 times in PBS and prepared for viewing by the addition of Vectashield mounting medium and a cover slip. The stained sections were viewed under white light to locate the area of gene gun inoculation marked by the presence of gold beads. These areas were then assessed for the presence of DC-like immune cells (FITC-labeled, anti-I-A^d^/I-E^d ^antibody positive cells with DC morphology) under fluorescent microscopy.

### Protein production, purification, and inoculations

The B2L ORF was amplified by PCR using D1701 strain DNA template and ligated in frame with a His6 tag-encoding sequence into the pQE60 vector (Qiagen Corp.) for expression and isolation of recombinant protein. The NZ-2 homolog was also amplified from genomic DNA (gift of Tobias Schlapp of Bayer Pharmaceuticals) and cloned into pQE60 for expression with a C-terminal 6-histidine (His6) tag. The yeast SRB4 gene was similarly cloned into pQE60 and produced as an irrelevant, similarly sized, His-tagged control protein. Plasmids were transformed into *E. coli *strain BL21, induced with 0.5 mM IPTG (Isopropyl β-D-1-thiogalactopyranoside) and cultured by standard protocols. The recombinant proteins were purified from bacterial extracts (B-Per, Pierce Chemical Co) using Ni-affinity chromatography and imidazole elution. Isolated proteins were dialyzed vs PBS + 10% glycerol. L and R designations are typically used to designate genomic positions of poxvirus genes, but not included in the nomenclature when the protein is indicated. Neither B2 nor the control protein samples contained detectable endotoxin by the amoebocyte lysate gelling assay. Hair was clipped from the site prior to protein injection. In initial tests, the recombinant protein was diluted with sterile PBS and injected intradermally (i.d.) into the abdomen at various concentrations ranging from 0.01-10 μg/ml, 50 μl/site. Inoculation sites were marked with India ink to facilitate subsequent tissue harvesting. A total dose of 50 ng/injection was found to be optimal for measuring cell accumulation. Tissues were harvested and assayed for DC recruitment as described following genetic inoculations.

### Serum antibody assay

To measure antibody response levels, blood was collected from each mouse at indicated time-points post immunization and assayed for antigen-specific antibodies by ELISA. Assay plates were coated with HBsAg (Seradyn, Inc., Indianapolis, IA) in PBS (10 ng/ml; 50 μl/well) overnight at 4°C. After the antigen solution was removed, plates were washed 3 times, blocked with 1% BSA in wash buffer and incubated with diluted mouse sera. The plates were washed and antigen-bound antibodies were incubated with goat anti-mouse-Ig-HRP conjugate (BioRad, Inc.). Plates were washed again and developed with 100 μl TMB substrate solution. HRP reactions were stopped by addition of an equal volume of 0.25 N HCl and absorbance at 450 nm was determined for each well. These absorbance values were plotted against a standard curve. The standard curve was derived by measuring reactivity of a dilution series of know amounts (ng/ml) of a commercially available murine monoclonal anti-HBsAg antibody (Seradyn, Inc.).

### Influenza challenge-protection assay

The influenza challenge experiment was conducted as part of a cooperative agreement with the University of New Mexico, supervised by C. Rick Lyons. Female BALB/c mice 4-6 weeks of age were purchased from Charles Rivers Laboratories (Wilmington, MA) and housed in a pathogen free environment at the University of New Mexico animal facility for at least 2 weeks before starting the experiments. All mice were maintained and cared for according to the guidelines of the institutional Animal Care and Use Committee. Mice (10 per group) were immunized twice (weeks 0 and 3) with plasmid encoding influenza hemagglutinin, pCMVi-HA (A/PR/8/34) [45]. Groups of 8 mice were immunized with one of four doses (0.4, 4 ng, 40 ng, 400 ng) of pCMVi-HA plasmid and either 1 μg control plasmid (pUC119), pCMVi-GMCSF, or pCMVi-B2L (D1701 strain). DNA doses are given per mouse; however, DNA was delivered in 2 shots of half-dose to each ear. For viral challenge (6 weeks post-boost) isofluorane-anesthetized mice were intranasally administered (in 50 μl PBS drops) 5 LD_50 _(= 2.4 × 10^4 ^50% egg-infecting dose, EID_50_) of mouse adapted influenza A/PR/8/34 (H1N1) (Advanced Biotechnology Inc.), as previously described [45]. The mice were monitored daily for 23 days post-challenge and mortality was recorded.

### Tumor protection assay

The effect of B2L on tumor development was evaluated in a transplantable model of melanoma. On day 1, two groups of 20 C57BL/6 mice were subcutaneously (s.c.) injected with 5 × 10^4 ^of the murine melanoma B16-F10 cells (American Type Culture Collection, Rockville, MD). The B16-F10 cells were grown in Minimal Essential Medium (MEM; Gibco, Rockville, MD), supplemented with 2 mM L-glutamine, 25 mM HEPES, 2.2 mg/ml sodium bicarbonate containing 10% FBS (Gibco), 100 U/ml penicillin, 100 μg/ml streptomycin, and 0.11 mg/ml sodium pyruvate solution (all from Sigma, St. Louis, MO). The cells were cultured in a humidified, 5% CO_2 _atmosphere at 37°C. On day 2, the test group was biolistically administered pCMVi-B2L as 4 shots of gene gun microparticles carrying 1 μg of DNA each (Helios, BioRad, Inc.). Tumor diameters were measured with a micronomer in triplicate by two individuals at indicated days post tumor cell injection. At day 13, mice began to be euthanized due to overly large tumors. Survival was monitored until day 25, and then all survivors were sacrificed. This experiment was repeated in this C57BL/6 transplantable melanoma model and also conducted in a BALB/c transplantable breast tumor model of cancer.

### Animal protocols

For the ORFV DNA and protein injections and for the influenza challenge-protection experiments six- to eight-week old female BALB/c mice were purchased from Charles River Laboratories, San Diego, CA. For the tumor -protection studies six- to eight-week old female C57BL/6 mice were purchased from Harlan Sprague Dawley, Indianapolis, IN. All mice were housed in a pathogen-free environment at either the University of Texas Southwestern Medical Center or the University of New Mexico. Mice were given food and water *ad libitum*, and all procedures were conducted under protocols approved by the Institutional Animal Care and Use Committees and in accordance with federal guidelines for animal experimentation.

### Statistical methods

Unpaired Students t tests were used to compare the antibody levels measured in the ELISA experiments and also to compare the tumor sizes of treated and untreated mice. The relatively small sample sizes (5 to 20 mice per group) and two-way comparisons of these experiments indicate the appropriateness of the t test, although other less stringent methods might be applied. The arithmetic mean is calculated for each group and the standard deviation (SD) used as a measure of variance, these are indicated by error bars for each plot. For the survival experiments, Kaplan-Meier survival curves were derived and the associated parametric test provided by the PRISM software package (Graphpad).

## List of abbreviations

LEE: linear expression element; Interferon-γ: γ-IFN; ORFV: *Parapoxvirus ovis; *LUC: luciferase; AAT: α-1 antitrypsin; MHC: major histocompatibility; ORF: open-reading frame; HA: hemagglutinin; DC: dendritic cells; HBsAg: hepatits B virus surface antigen.

## Competing interests

SAJ and MJM were issued a patent for certain aspects of the work presented here (US 6,752,995). KFS declares no competing interests.

## Authors' contributions

MJM, SAJ, and KFS contributed to the conception of the screening approach, design of the experiment, and analysis of the results. MJM carried out all studies. KFS wrote the manuscript, with input from both MJM and SAJ. All authors read and approved the final version.

## Supplementary Material

Additional file 1 **The B2 protein of ORFV strain NZ2 also displays immune-cell accumulating activity following murine skin inoculation**. Mice were injected i.d into the abdomen with PBS (**left panel**) or 50 ng rB2 protein (**right panel**) in triplicate. After 4 days, immune cell densities were measured in harvested skin by 2G9 immunostaining, as described in Figures 2 and 3. Scale bars are 40 micron. Click here for file

Additional file 2 **No detectable antibody response to B2 is elicited by mice co-immunized with rHBsAg and rB2**. Sera from the same groups of mice (4 to 5 per group) assayed for HBsAg-reactivity in Figure 4b were assayed for reactivity to B2. The ELISA was conducted in duplicate as described in Material and Methods and Figure 4b except plates were coated with rB2 (50 μl/well) instead of HBsAg. Click here for file
