# Supplementary figures and images for: Novel immune-modulator identified by a rapid, functional screen of the parapoxvirus ovis (Orf virus) genome
Source: Proteome Sci. 2012 Jan 13;10:4. doi: 10.1186/1477-5956-10-4 (PMC3283511; doi:10.1186/1477-5956-10-4)

## Slide 1
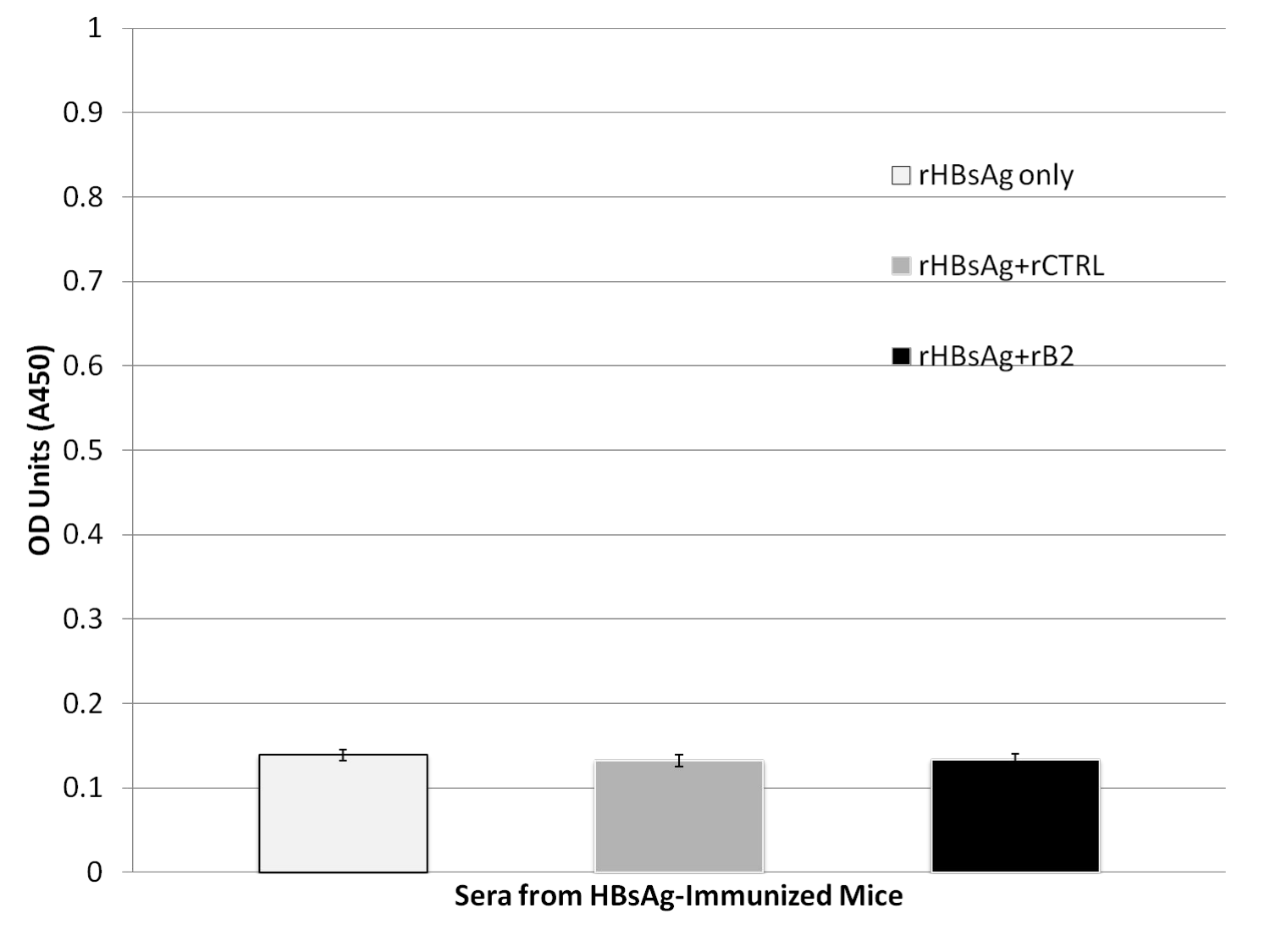

Supplement: Additional file 2 — No detectable antibody response to B2 is elicited by mice co-immunized with rHBsAg and rB2. Sera from the same groups of mice (4 to 5 per group) assayed for HBsAg-reactivity in Figure 4b were assayed for reactivity to B2. The ELISA was conducted in duplicate as described in Material and Methods and Figure 4b except plates were coated with rB2 (50 μl/well) instead of HBsAg. [file 1477-5956-10-4-S2.PPT]
